# Contribution of silicon fertilizer to soil and growth of Pak choi under reclaimed and brackish water cycling irrigation

**DOI:** 10.1371/journal.pone.0322846

**Published:** 2025-05-07

**Authors:** Jieru Zhao, Bingjian Cui, Juan Wang, Qibiao Han, Chao Hu, Rui Li, Chuncheng Liu

**Affiliations:** 1 Institute of Farmland Irrigation, Chinese Academy of Agricultural Sciences, Xinxiang, China; 2 College of Hydraulic Science and Engineering, Yangzhou University, Yangzhou, China; 3 Agriculture Water and Soil Environmental Field Science Research Station of Xinxiang City of Chinese Academy of Agricultural Sciences, Xinxiang, China; Nuclear Science and Technology Research Institute, IRAN, ISLAMIC REPUBLIC OF

## Abstract

Rational utilization and improvement of agricultural water resources has been and is still the focus of research on developing efficient and green agriculture in various countries. Thus, the exploitation and usage of non-traditional water resources hold substantial significance in water resources management and sustainable agriculture. However, their reuse may induce secondary soil salinization and impose stress on crops. To address the challenges of soil salinity and plant stress under brackish-reclaimed water irrigation, this study aimed to investigate the effects of silicon (Si) fertilizer application on soil properties and Pak choi (*Brassica rapa* L.) performance under two cycling irrigation sequences (RW-BW and RW-RW-BW) and three spraying frequencies (0-, 2-, and 4-day intervals). The findings displayed that the pH of each treatment (7.95-8.10) remained below 8.5, suggesting no risk of secondary soil alkalization. At the same spraying frequency of silicon fertilizer, the soil electrical conductivity (EC) significantly decreased with increasing irrigation times of reclaimed water. Silicon fertilizer improved soil structure and reduced sodium levels, alleviating salinity. The increasing spraying interval of silicon fertilizer provoked the diminution of the SAR and ESP, before rising again. But they were far below the threshold range, and there was no risk of soil salinization (15% and 13 mM^1/2^). The total silicon content of the soil and leaves increased under the different cycling irrigation conditions. Spraying silicon fertilizer on the crop leaf surface did not significantly influence the total silicon content of the soil. In conclusion, the application of Si-fertilizer beneficially impacts soil physicochemical properties and crop development and mitigates the risk of secondary salinization under brackish-reclaimed water for cycling irrigation.

## Introduction

Water is a fundamental resource for human survival and various economic activities [1]. Enhancing the efficient use of water in agriculture and boosting water use efficiency is the research focus for developing efficient and green agriculture in various countries [2]. With the rapid economic development, the shortage of quality water has gradually become a major factor restricting the development of Chinese agriculture [3]. Therefore, the development and use of non-traditional water sources are highly important for alleviating agricultural water resource problems. According to the China Water Resources Bulletin from 2010 to 2022, the proportion of the total water supply from unconventional sources in China increased from 0.7% to 2.9%. Reusing water and enhancing water use efficiency can significantly relieve water deficits [4].

Agriculture has increasingly shifted toward unconventional water sources, such as reclaimed water (RW) and brackish water (BW). BW irrigation has become a strategy to sustain agricultural development, particularly in arid regions. Rational utilization of BW can effectively expand irrigation water resources [5], alleviate shortages of freshwater resources [6], and stimulate crop growth [7]. However, brackish water contains large amounts of Na^+^ and Cl^-^, that can increase soil bulk density [8], reduce soil porosity [9], hinder soil permeability [10], decrease permeability [11], reduce soil organic matter content [12], and cause soil water repellency [13], and the soil structure is further damaged. The soil salinization caused by irrigation has led to the degradation of agricultural areas. Currently, there are two common utilization methods, mixed irrigation and cycling irrigation together with fresh water. However, freshwater resources are greatly short in areas where brackish water is used. Therefore, these methods are often not easy to implement, and new approaches must be explored to utilize brackish water. RW is available in large quantities and maintains stable water quality, making it extensively utilized across various sectors globally, including industry, agriculture, municipalities, and landscaping [14–17]. The use of reclaimed water from treated urban domestic sewage for irrigation poses significantly lower pollution risks compared to irrigation with untreated wastewater. Additionally, reclaimed water is enriched in essential nutrients, which can help the growth of plants [18], enhance water sustainability, and improve resource efficiency [19], particularly in agriculture and industry. However, it has potential health risks due to contaminants, varying public acceptance, and the need for significant infrastructure investment [20]. Irrigation combined with reclaimed water and brackish water can help mitigate the adverse impacts of salt on crop growth, facilitate soil carbon accumulation [21], maintain nutrients in soil [22], and increase the concentration of exchangeable Ca^2+^ in soil [23]. While the combination of brackish and reclaimed water offers many benefits, it also introduces certain challenges. Since brackish water contains salts, long-term use carries the potential risk of secondary soil salinization, which may impact crop growth and yield. Consequently, appropriate agronomic measures are essential to mitigate this risk when using brackish and reclaimed water as cycling irrigation sources.

Pak choi (*Brassica rapa* L.), a cruciferous vegetable with high nutritional value and a short growth cycle, is widely cultivated due to its significant economic and market importance. Its extensive production supports farmer livelihoods, stabilizes vegetable supply, and meets consumer demand. Given these attributes, Pak choi was selected as the experimental material in this study to investigate the regulatory effects of silicon on soil and crop performance under reclaimed-brackish water cycling irrigation, aiming to provide a theoretical basis and technical reference for the sustainable use of unconventional water resources and stable crop production. Silicon fertilizer has received extensive attention as a substance with the function of salt stress regulation. Silicon (Si) occurs naturally in air, water, and soil [24–26] and is the second most prevalent element in the lithosphere, accounting for 26.3% of the total mineral content [27]. Rational utilization of Si-fertilizer has been shown to significantly enhance the photosynthetic performance of crops [28], promote the growth and symbiosis of beneficial microorganisms [29], promote the development of crop stems and roots [30], and improve plant tolerance to salinity stress [31]. Si is a vital nutrient for promoting plant growth, and development, and supporting various metabolic processes [32]. Numerous studies on the alleviating influences of exogenous silicon on plants under salt stress have primarily focused on growth, physiological, and biochemical responses. Silicon can enhance plant salt tolerance by regulating hormone expression and hormone-responsive genes [33,34] and mitigate salt-induced osmotic stress by modulating the biosynthesis and metabolism of aquaporins and osmoregulatory compounds. Exogenous silicon can effectively alleviate the related damage to photosynthetic pigments and photosynthetic capacity under salt stress, and it also reduces the damage to reactive oxygen species production and accumulation, to ensure normal plant growth [35,36].

At present, research on RW-BW mixed irrigation is mainly focused on soil water repellency [37], soil enzyme activity [38], soil characteristics [39], crop physiological characteristics [40], etc. Cycling irrigation (CI) [41] is one of the most effective methods for utilizing BW. However, studies examining the impacts of silicon fertilizer on soil and crops are still limited under cycling irrigation with brackish-reclaimed water. The use of exogenous silicon has been previously neglected. Thus, this study aimed to investigate the impact of exogenous silicon on soil properties and the growth of Pak choi when irrigated with cycling brackish and reclaimed water. It provides new methods to enhance agricultural productivity, promotes the development of eco-agriculture, and lays the groundwork for future research, advancing the integration of soil science and plant physiology.

## Materials and methods

### Test materials

The experiment was conducted in a sunlight greenhouse (day/night temperature,32 °C/20 °C; relative humidity, 50–60%) of the Agriculture Water and Soil Environmental Field Science Research Station of Xinxiang City of Chinese Academy of Agricultural Sciences, which is an institutional research facility owned and operated by Chinese Academy of Agricultural Sciences, and no external permits were required for access. The tested soil was collected from nearby farmland, air-dried, ground, and sieved through a 2-mm mesh for subsequent use. The soil had a bulk density of 1.40 g·cm^-3^, a field capacity of 17.27% by mass, an electrical conductivity of 264 μS·cm^-1^ in a 1:5 soil–water extract, and a soil organic matter of 2.31%. The soil texture is a loam (international system) due to 20.90% in clay, 44.62% in silt, and 34.48% in sand by a BT-9300HT laser particle size analyzer.

The pots used in the experiment had a top diameter of 250 mm, a bottom diameter of 145 mm, and a height of 190 mm. Each pot contained 7 kilograms of soil and was laid out randomly. The “Pak choi”, often called Shanghai Green, was selected as the experimental plant, and the seeds were sown evenly on June 9, 2021. A 7 g dose of compound fertilizer (with an N-P_2_O_5_-K_2_O ratio of 15-15-15) was applied as base fertilizer to each pot according to the local recommendations. On June 21, the plants reached the two-leaf, one-heart stage, each pot was thinned to five plants, and the experiment officially commenced. Watering was provided in 300 mL increments whenever soil moisture, monitored using a portable soil moisture meter, dropped below 75% of field capacity. Throughout the trial, management practices (such as irrigation amount, irrigation method, agronomic measures, etc.) were maintained consistently.

### Experimental design

In this experiment, two factors were established: cycling irrigation sequence (CS) and silicon fertilizer spraying frequency (SFSF). The cycling irrigation sequence included two levels: reclaimed-brackish water, and reclaimed-reclaimed-brackish water. The spraying frequency of foliar silicon fertilizer was set at two levels—application every 2 days and every 4 days, with a control group that received no silicon fertilizer. The concentration of silicon fertilizer (sodium metasilicate) was maintained at 150 mg·L^-1^ according to the existing research result [42]. A total of six treatments were applied, each with three replicates, as detailed in Table 1. The quality of the different water sources is presented in Table 2. Reclaimed water (RW) used in this study was obtained from the Luotuowan Domestic Sewage Treatment Plant in Xinxiang City, Henan Province. This water had undergone A^2^/O treatment and complied with the “Water Quality Standard for Farmland Irrigation” (GB5084–2021). The BW was prepared by blending sea salt into the freshwater according to the research results of Zhang et al. [43].

**Table 1:** Experimental treatments and design for cycling irrigation sequence (CS) and silicon fertilizer spraying frequency (SFSF).

CS level	SFSF level/d	Treatment
RW-BW	0(no spraying)	RB0
	2	RB2
	4	RB4
RW-RW-BW	0(no spraying)	RRB0
	2	RRB2
	4	RRB4

Note: “RW” means Reclaimed water; “BW” means Brackish water; “RW-BW” means “Reclaimed-brackish water” cycling irrigation; “RW-RW-BW” means “Reclaimed-reclaimed-brackish water” cycling irrigation.

**Table 2:** Physicochemical properties and ion concentrations of reclaimed water (RW) and brackish water (BW).

Water Resource	EC (μS·cm^-1^)	pH	Na^+^ (mmol·L^-1^)	K^+^ (mmol·L^-1^)	HCO_3_^-^ (mmol·L^-1^)	Cl^-^ (mmol·L^-1^)	Ca^2+^ (mmol·L^-1^)	Mg^2+^ (mmol·L^-1^)	SO_4_^2-^ (mmol·L^-1^)	SAR (mmol·L^-1^)^1/2^	TN (mg·L^-1^)	TP (mg·L^-1^)	Pb (mg·L^-1^)	Cu (mg·L^-1^)	Zn (mg·L^-1^)	Cd (mg·L^-1^)
RW	2120	8.17	13.5	0.36	4.56	8.85	2.28	3.10	5.28	5.81	0.52	0.05	ND	ND	ND	ND
BW (5 g·L^-1^)	9432	8.44	87.0	0.07	2.28	90.90	0.92	0.77	1.14	66.86	1.18	0.02	ND	ND	ND	ND

Note: “RW” means Reclaimed water; “BW” means Brackish water. ND signifies not detected.

### Determination index and method

(1) Soil physical and chemical properties analysis. The soil moisture was measured using the gravimetric drying method. Soil pH was measured using a potentiometric method (soil-to-water ratio 1:2.5). Electrical conductivity (EC, soil-to-water ratio 1:5) was assessed by conductivity meter (DDB-303A), water-soluble Na^+^ and K^+^ concentrations were determined by flame photometry (FP6410), Ca^2+^ and Mg^2+^ concentrations were quantified via EDTA titration, Cl^–^ was determined by AgNO_3_ titration, CO₃² ⁻ and HCO_3_^–^ were analyzed by double indicator neutralization titration, and SO₄² ⁻ was measured using indirect complexometric titration with EDTA [39]. The sodium adsorption ratio (SAR) was calculated from water-soluble Na ⁺ , Ca² ⁺ , and Mg² ⁺ concentrations [44]. The soil organic matter (SOM) was evaluated using low-temperature, external thermal potassium dichromate oxidation colorimetry [39]. Water repellency was assessed by measuring the water drop penetration time (WDPT) [45]. Exchangeable K ⁺ and Na⁺ concentrations were determined via NH₄Cl-ethanol exchange followed by flame photometry (FP6410), and exchangeable Ca² ⁺ and Mg² ⁺ were measured using NH₄Cl-ethanol exchange coupled with atomic absorption spectrophotometry (AA7000F). The exchangeable sodium percentage (ESP) was calculated as the ratio of exchangeable Na ⁺ to cation exchange capacity (CEC), determined via the Ca(OAc)₂ method.
(2) Soil enzyme activity. Soil alkaline phosphatase (S-AKP/ALP) activity was measured using a detection kit (Solarbio, Beijing), where one enzyme activity unit corresponds to the release of 1 nmol of phenol per gram of soil per day at 37°C. Soil sucrase (S-SC) activity was measured by the 3,5-dinitrosalicylic acid colorimetric method, with results expressed as the amount of glucose (mg) produced per gram of soil over 24 hours. Urease (S-UE) activity was evaluated using indophenol colorimetry, following the method of Guan [46]. In this procedure, a culture mixture was added to the outer ring of the dish, while 3%H_3_BO_3_ and a mixed indicator methyl red and bromocresol green were placed in the inner ring, and then incubated at 37°C. Released NH_3_-N was measured daily or every other day by titration with standard H_2_SO_4_.
(3) Crop biomass. Following harvest, plants were rinsed with distilled water, and fresh weights of aboveground (AFW) and underground (UFW) parts were recorded using an analytical balance. The plant samples were then oven-dried at 105°C for 15 minutes and subsequently oven-dried to constant weight at 75°C. This process enabled the determination of the dry weights of the aboveground (ADW) and underground (UDW) components using a balance.
(4) Crop antioxidant properties. Following harvest, the collected plant tissue samples were frozen in liquid nitrogen, and then ground into fine powder in liquid nitrogen after being brought back to the laboratory. Phosphate buffer was used to mix the ground powder evenly at a ratio of 1:10. After the shock, the supernatant was centrifuged and the activity of antioxidant enzymes was determined. The chlorophyll a, chlorophyll b, and total chlorophyll contents in leaves were measured using a detection kit (Soleibao, Beijing). The soluble protein content was determined using the Coomassie brilliant blue method at 595nm. Additionally, the activities of catalase (CAT), superoxide dismutase (SOD), and peroxidase (POD) were measured employing the UV absorption method, the nitrogen blue tetrazolium photochemical reduction method, and the guaiacol method at wavelengths of 240 nm, 560 nm, and 470 nm, respectively [22]. Malondialdehyde (MDA) content was quantified using the thiobarbituric acid (TBA) method at 452 nm, 532 nm, and 600 nm wavelengths [22].
(5) Total silicon concentration. The total silicon concentration in the soil was determined using the animal glue desilication - mass method. The procedure involved melting the sample with sodium carbonate and dissolving it in hydrochloric acid. The resulting solution was then evaporated to a damp salt state. In a concentrated hydrochloric acid medium, animal glue was added to promote the condensation of silicic acid, which was subsequently dehydrated by silica precipitation. The precipitate was filtered to separate from other elements, calcined at 920°C, and weighed to determine the SiO_2_ content. Finally, the silicon content was calculated by converting the SiO_2_ weight [47].

Furthermore, the leaf samples were digested with a mixture of HNO_3_ and HClO_4_, and the SiO_2_ extracted from the digestate was then calcined at 800°C. The silicon concentration in the leaves was subsequently quantified using the mass method [48].

### Data analysis

Two-way ANOVA was conducted by SPSS 25.0 software (IBM Crop., Armonk, NY, USA), and pairwise comparisons of the samples were carried out with the LSD test at 0.05 level. Origin 8.0 (OriginLab Corporation, Northampton, MA, USA, 2007) was utilized to draw figures.

## Results

### Regulation of soil by Si-fertilizer under cycling irrigation(CI)

#### Physical-chemical characteristics of soil.

(1) Soil Moisture and Salinity

The changes in the soil moisture and EC after harvest in all treatments are shown in Table 3.

**Table 3:** Soil moisture and EC under cycling irrigation (CI) combined with Si fertilization.

Treatment	Soil Moisture Content (%)	EC/(μS·cm^-1^)
RB0	16.46 ± 0.71a	747.67 ± 1.53b
RB2	16.21 ± 0.40ab	789.00 ± 5.29a
RB4	16.46 ± 0.14a	786.00 ± 2.65a
RRB0	14.94 ± 1.03b	702.33 ± 2.52d
RRB2	15.03 ± 0.25b	720.67 ± 1.53c
RRB4	15.08 ± 0.12b	692.67 ± 0.58e
LSD_0.05_	0.9550	4.8102
CS	**	**
SFSF	ns	**
CS *SFSF	ns	**

Note: Data are presented as means ± standard errors. Different lowercase letters on the column indicate significant differences by LSD test (*P* < 0.05). *, ** represent significant differences at the levels of 0.05 and 0.01, respectively; ns represents no significant difference. RB0 stands for the reclaimed-brackish water alternating irrigation and no silicon fertilizer, RB2 stands for the reclaimed-brackish water alternating irrigation and silicon fertilizer spray every 2 days, RB4 stands for the reclaimed-brackish water alternating irrigation and silicon fertilizer spray every 4 days, RRB0 stands for the reclaimed-reclaimed-brackish water alternating irrigation and no silicon fertilizer, RRB2 stands for the reclaimed-reclaimed-brackish water alternating irrigation and silicon fertilizer spray every 2 days, RRB4 stands for the reclaimed-reclaimed-brackish water alternating irrigation and silicon fertilizer spray every 4 days. “CS” means cycling irrigation sequence; “SFSF” means silicon fertilizer spraying frequency.

As shown in Table 3, the results of ANOVA indicated that CS, SFSF, and their interaction all had great significant effects on EC (*P* < 0.01). Under cycling irrigation with BW and RW, no significant difference in soil moisture was observed among treatments as SFSF was extended. In addition, when the silicon fertilizer spraying frequency was constant, soil moisture exhibited a decreasing trend as the cycling irrigation time of reclaimed water increased, and no notable difference occurred between treatments when the spraying frequency was 2 days. The results of ANOVA showed that CS had a great significant effect on soil moisture content (*P* < 0.01), while the SFSF and their interaction had no significant effects. The EC in all the soils treated with alternative irrigation varied between 692.67 and 789 μS·cm^-1^. The EC in the treatments with silicon fertilizer significantly increased by 5.13%-5.53% compared with the treatments without silicon fertilizer under the “reclaimed-brackish water” cycling irrigation. The EC in the treatments with silicon fertilizer decreased significantly by 1.38% compared with the no-spraying silicon fertilizer treatment under the “reclaimed-reclaimed-brackish water” cycling irrigation. Furthermore, the EC decreased significantly with increasing irrigation times of reclaimed water when the silicon fertilizer spraying frequency was constant.

(2) Soil water-soluble ions

**Fig 1:**
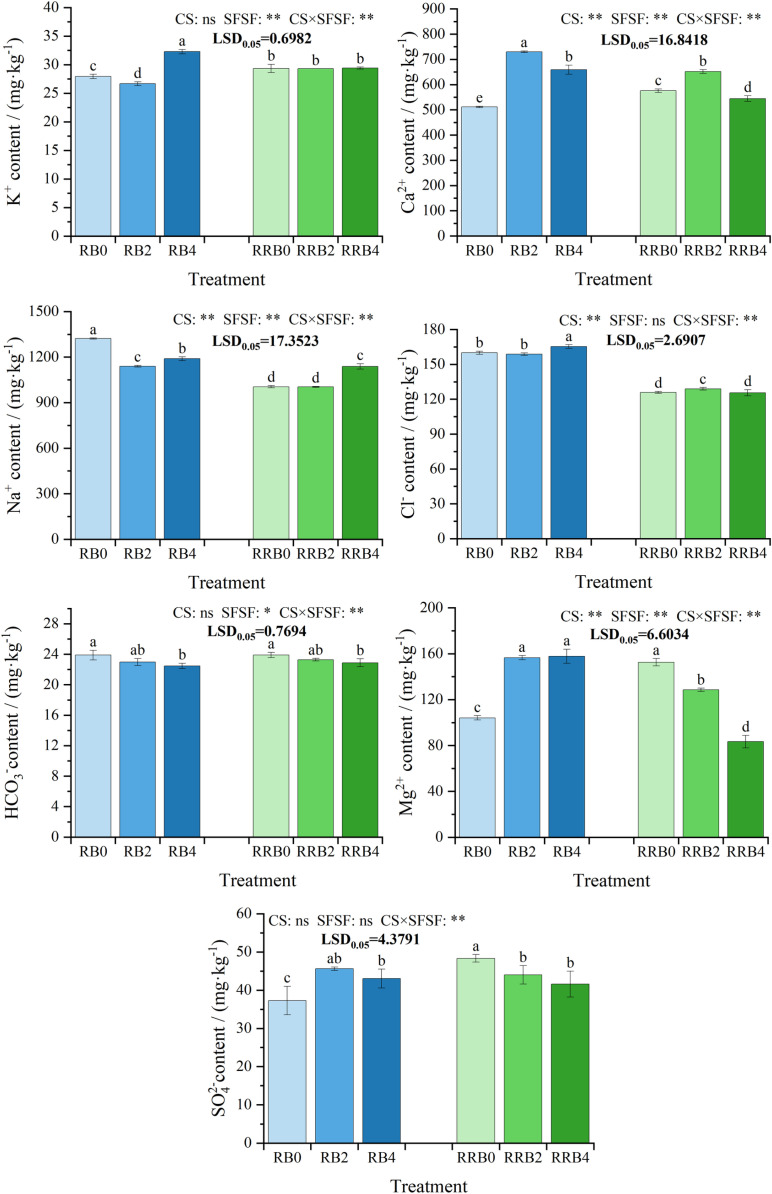
The interaction effect of CS*SFSF on soil ion content under cycling irrigation (CI) combined with Si fertilization. Data are presented as means ± standard errors. Different lowercase letters on the column indicate significant treatment differences by LSD test (*P* < 0.05) *, ** represents a significant difference at the levels of 0.05 and 0.01, respectively; ns represents no significant difference.

The changes in soil water-soluble ions contents after harvest under different spraying frequencies of silicon fertilizer and cycling irrigation modes are shown in Fig 1.

As illustrated in Fig 1, according to the results of ANOVA, CS had no significant effects on K^+^, HCO_3_^-^, and SO_4_^2**-**^ contents, while it had significant effects on other water-soluble ions (*P* < 0.05). SFSF had no significant effects on Cl^-^, SO_4_^2**-**^ content, while it had significant effects on other water-soluble ions (*P* < 0.05). Furthermore, their interaction had no significant effects on HCO_3_^-^ content, while it had significant effects on other water-soluble ions (*P* < 0.05).

1) for the soil K^+^ and Ca^2+^ content, compared with the treatment without Si-fertilizer, the highest K^+^ and Ca^2+^ content of the soil was found in the treatment with spraying frequency for 4 and 2 days, respectively, and the differences in K^+^ (only under the “reclaimed-brackish water” alternative irrigation) and Ca^2+^ between treatments reached a significant level.
2) In terms of the soil Na^+^ and Cl^-^ contents, treatment involving the application of silicon fertilizer every two days led to notably lower soil Na^+^ levels compared to treatments without silicon (Si) fertilization under RW-BW cycling irrigation. Under RW-BW cycling irrigation, the soil chloride (Cl^-^) content was minimized when silicon fertilizer was sprayed every two days. Meanwhile, under the RW-RW-BW cycling irrigation, the lowest soil Cl^-^ level was observed following silicon fertilizer application every four days. However, these differences in Cl^-^ content between the treatments did not achieve statistical significance.
3) Compared with scenarios without any silicon (Si) fertilization, after spraying silicon fertilizer, the HCO_3_^-^ content in soil showed a gradually decreasing trend with significant differences observed at the 4-day spraying frequency, the soil Mg^2+^ content significantly increased under the “ RW-BW “ cycling irrigation while significantly decreased under the “RW-RW-BW” cycling irrigation, and the change trend of the soil SO_4_^2-^ content was consistent with that of soil Mg^2+^ content.

(3) Soil organic matter (SOM) content and WDPT.

**Fig 2:**
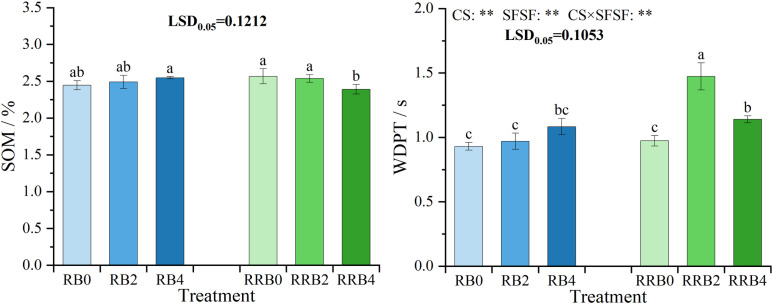
The interaction effect of CS*SFSF on SOM and WDPT under cycling irrigation (CI) coupled with Si fertilization. Data are presented as means ± standard errors. Different lowercase letters on the column indicate significant treatment differences by LSD test (*P* < 0.05). *, ** represents a significant difference at the levels of 0.05 and 0.01, respectively; ns represents no significant difference.

Fig 2 illustrates the variations in SOM and WDPT. The results of ANOVA showed that CS, SFSF, and their interaction had significant effects (*P* < 0.01) on WDPT. As depicted, the application of silicon fertilizer under the RW-BW cycling irrigation increased the SOM content, although the differences among treatments were not statistically significant. Under the RW-RW-BW cycling irrigation, however, SOM content decreased, with no significant difference observed between RRB0 and RRB2 treatments. Furthermore, all treatments recorded WDPT values well below 5 seconds, indicating the absence of soil water repellency.

#### Soil enzyme activity.

Table 4 shows the changes in alkaline phosphatase (AKP/ALP), soil sucrase (S-SC), and soil urease (S-UE) activities under different treatments. Based on the results from ANOVA, CS had no significant effects on AKP/ALP activity, S-SC activity, or S-UE activity. SFST had no significant effects on AKP/ALP activity and S-SC activity, while it had a significant effect on S-UE activity (*P* < 0.01), and their interaction had no significant effects on S-SC activity and S-UE activity, while it had a significant effect on AKP/ALP activity(*P* < 0.05).

**Table 4:** Soil enzyme activity under CI combined with Si fertilizer.

Treatment	AKP/ALP activity/(U·g^-1^)	S-SC activity/(mg·g^-1^·24 h^-1^)	S-UE activity/(mg·g^-1^·24 h^-1^)
RB0	3056.37 ± 1271.98ab	8.6 ± 0.08ab	0.54 ± 0a
RB2	3091.91 ± 1537.66ab	8.24 ± 0.19b	0.52 ± 0.01b
RB4	2096.81 ± 162.86b	8.54 ± 0.14ab	0.53 ± 0a
RRB0	1030.64 ± 123.11b	8.66 ± 0.09a	0.54 ± 0a
RRB2	2416.67 ± 123.11b	8.34 ± 0.44ab	0.52 ± 0.01b
RRB4	4122.55 ± 325.72a	8.41 ± 0.09ab	0.54 ± 0.01a
LSD_0.05_	1438.7683	0.3686	0.0071
CS	ns	ns	ns
SFSF	ns	ns	**
CS *SFSF	*	ns	ns

Note: Data are presented as means ± standard errors. Different lowercase letters on the column indicate significant treatment differences by LSD test (*P* < 0.05). *, ** represent significant differences at the levels of 0.05 and 0.01, respectively; ns represents no significant difference. RB0 stands for the reclaimed-brackish water alternating irrigation and no silicon fertilizer, RB2 stands for the reclaimed-brackish water alternating irrigation and silicon fertilizer spray every 2 days, RB4 stands for the reclaimed-brackish water alternating irrigation and silicon fertilizer spray every 4 days, RRB0 stands for the reclaimed-reclaimed-brackish water alternating irrigation and no silicon fertilizer, RRB2 stands for the reclaimed-reclaimed-brackish water alternating irrigation and silicon fertilizer spray every 2 days, RRB4 stands for the reclaimed-reclaimed-brackish water alternating irrigation and silicon fertilizer spray every 4 days. “CS” means cycling irrigation sequence; “SFSF” means silicon fertilizer spraying frequency.

As shown in Table 4, the AKP/ALP activity in the treatment with a 2-day spraying frequency was greater than that in the treatment without silicon fertilizer under different cycling irrigation modes, although their difference did not reach statistical significance. The S-SC activity exhibited no significant differences between the treatments with and without spraying silicon fertilizer, regardless of the irrigation mode. No statistically significant difference was observed in S-UE activity between the treatment with a 4-day spraying frequency and the treatment without spraying, while the S-UE activity in the 2-day spraying frequency treatment was significantly lower than that in the treatment without spraying.

#### Potential for secondary soil salinization.

**Fig 3:**
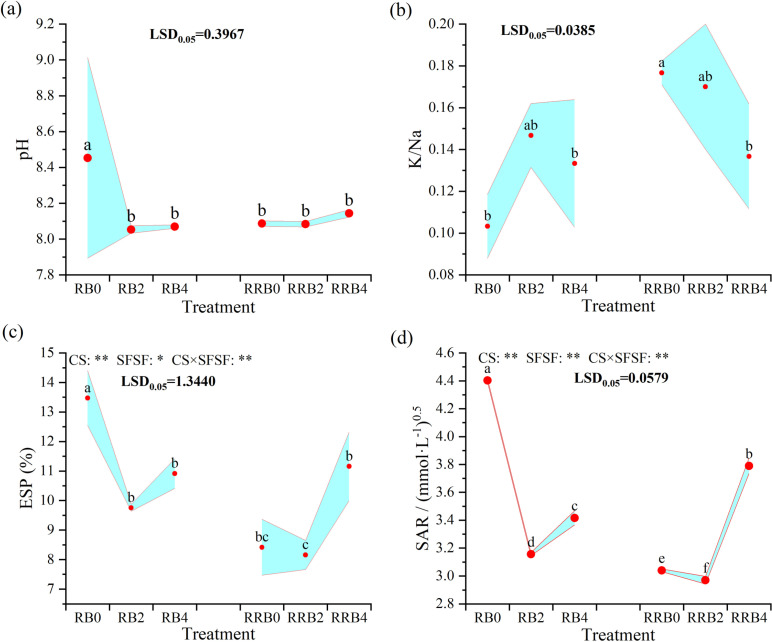
The interaction effect of CS*SFSF on Changes in soil pH (a), potassium to sodium ratio (K/Na) (b), ESP (c), and SAR (d) under cycling irrigation (CI) coupled with silicon fertilization. Data are presented as means ± standard errors. Different lowercase letters on the column indicate significant treatment differences by LSD test (*P* < 0.05). *, ** represents a significant difference at the levels of 0.05 and 0.01, respectively; ns represents no significant difference.

The changes in the soil secondary salinization indices (pH, exchangeable K/Na, SAR, and ESP) under different cycling irrigation modes coupled with different silicon fertilizer application frequencies are shown in Fig 3.

As shown in Fig 3, all treatments had a pH between 7.95 and 8.10, which was below the threshold of 8.5, suggesting no threat of alkaline conditions in the soil. The pH levels were notably lower in the silicon-fertilized treatments compared to the untreated ones when the RW-BW cycling irrigation method was employed. However, under “reclaimed-reclaimed-brackish water” cycling irrigation, the pH of the treatments with silicon fertilizer and those without did not significantly change, and the pH was slightly lower with a 2-day silicon fertilizer spraying frequency. In contrast, under the RW-RW-BW cycling irrigation, the pH difference between treatments with and without silicon fertilizer was insignificant, with a slight decrease observed at the 2-day spraying frequency with silicon fertilizer.

Under “reclaimed-brackish water” cycling irrigation, the soil potassium-to-sodium (K/Na) ratio in pots treated with silicon fertilizer was slightly higher than those without silicon fertilizer, though the difference was not statistically significant. However, the soil K/Na in silicon fertilizer treatments decreased compared with no silicon fertilizer treatment under “reclaimed-reclaimed-brackish water” cycling irrigation, and the difference did not reach a significant level when the spraying frequency was 2 days. A higher K/Na ratio suggests better soil health and plant resilience, as potassium is essential for plant growth while sodium can be toxic. An appropriate increase in silicon fertilizer had a positive effect on the soil K/Na ratio under the “reclaimed-brackish water” cycling irrigation. Under the RW-RW-BW cycling irrigation, however, the soil K/Na ratio in silicon-fertilized treatments decreased compared to untreated pots, with the difference remaining insignificant when the silicon fertilizer was applied every two days. These findings suggest that an appropriate application of silicon fertilizer can beneficially influence the soil K/Na ratio under the “reclaimed-brackish water” cycling irrigation.

According to the analysis of ANOVA, CS, SFSF, and their interaction all had significant effects on ESP and SAR (*P* < 0.05). The soil ESP and SAR ranged from 8.16% to 13.47% and 2.97 to 4.40 (mmol/L)^1/2^, respectively, which were far below the threshold (15% and 13 mM^1/2^), posing no risk of soil salinization. Under the “reclaimed-brackish water” cycling irrigation, both the ESP and SAR in the silicon fertilizer-treated pots were significantly lower than those without silicon fertilizer, with decreases of 18.98%-27.58% and 22.41%-28.31%, respectively. Under the RW-RW-BW cycling irrigation, the ESP and SAR in the treatment of 2 d spraying frequency were lower compared to no silicon fertilizer treatment, which decreased by 3.09% and 2.30%, respectively. Both the soil ESP and SAR exhibited similar tendencies, namely, they both decreased first and then increased with the increasing spraying frequency, and both reached their lowest values when the silicon fertilizer spraying frequency was 2 days.

### Regulation of crops by Si-fertilizer under CI

#### Biomass.

**Fig 4:**
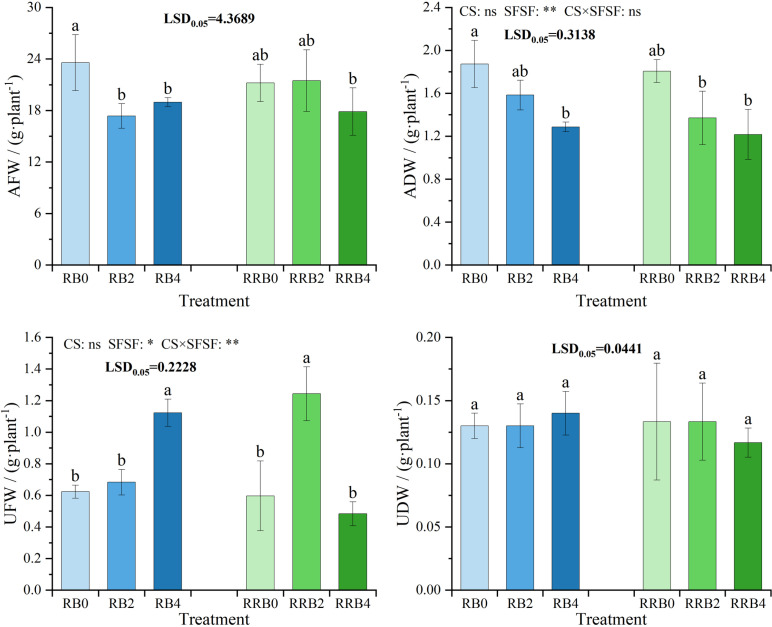
The interaction effect of CS*SFSF on fluctuations in Pak choi biomass under cycling irrigation (CI) paired with Si fertilization. Data are presented as means ± standard errors. Different lowercase letters on the column indicate significant treatment differences by LSD test (*P* < 0.05); *, ** represents a significant difference at the levels of 0.05 and 0.01, respectively; ns represents no significant difference.

The changes in biomass of the aboveground and underground parts of Pak choi during different treatments are shown in Fig 4. The results of ANOVA showed that both CS and the interaction had no significant effects on ADW, while SFSF had a significant impact on ADW (*P* < 0.05). As depicted, the application of silicon fertilizer led to a decrease in both AFW and ADW compared to scenarios without silicon fertilizer. This reduction was statistically significant at a spraying frequency of every 4 days under the “reclaimed-brackish water” cycling irrigation. Interestingly, under “reclaimed-reclaimed-brackish water” alternate irrigation, there were no substantial differences observed in AFW and ADW between treatments with and without silicon fertilizer application; however, it is worth noting that the AFW exhibited a slight increase at a spraying frequency of every 2 days compared to the no-spray treatment. These findings underscore the importance of optimal silicon fertilizer usage, which has a potential to enhance Pak choi yield, as evidenced by the increase in AFW under appropriate application conditions.

The results of ANOVA indicated that CS had no significant effect on UFW, while SFSF and the interaction had a significant effect on UFW (*P* < 0.05). The application of silicon fertilizers led to increased UFW and UDW compared to untreated controls. The greatest improvements were observed with a 4-day spraying frequency under the “reclaimed-brackish water” cycling irrigation. However, the UFW and UDW were generally highest with a 2-day spraying frequency under “reclaimed-reclaimed-brackish water” cycling irrigation. These findings underscore the role of precise silicon fertilizer management in enhancing the biomass of underground plant parts.

#### Indices of crop physiology and biochemistry.

(1) Leaf chlorophyll concentration

**Fig 5:**
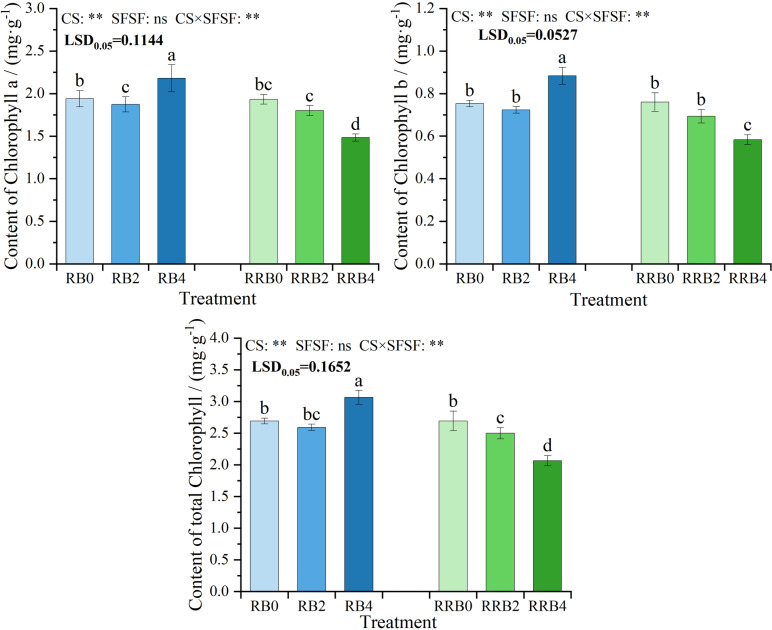
The interaction effect of CS*SFSF on Chlorophyll levels subjected to varied cycling irrigation (CI) modes coupled with silicon supplementation. Data are presented as means ± standard errors. Different lowercase letters on the column indicate significant treatment differences by LSD test (*P* < 0.05); *, ** represents a significant difference at the levels of 0.05 and 0.01, respectively; ns represents no significant difference.

Fig 5 illustrated the fluctuations in chlorophyll a, b, and total chlorophyll content in leaves subjected to various treatment conditions. The results of ANOVA indicated that CS and the interaction had significant effects on chlorophyll a, chlorophyll b, and total chlorophyll content in leaves (*P* < 0.05), while SFSF had no significant effects on them. As depicted, the trends of chlorophyll a, chlorophyll b, and total chlorophyll content were consistent. These chlorophyll contents first decreased and then increased with the increasing silicon fertilizer spraying frequency under “reclaimed-brackish water” cycling irrigation, and the highest levels were reached at the spraying frequency of 4 days. In contrast, all three chlorophyll concentrations steadily decreased with the increasing spraying frequency under RW-RW-BW cycling irrigation.

(2) Leaf antioxidant performance

**Fig 6:**
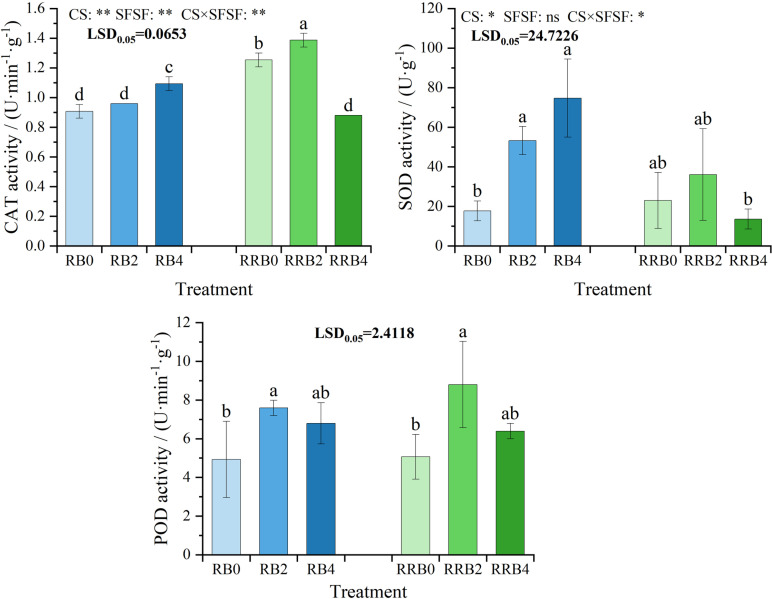
The interaction effect of CS*SFSF on enzyme Activity Fluctuations in Leaves under cycling irrigation (CI) combined with Si fertilization. Data are presented as means ± standard errors. Different lowercase letters on the column indicate significant treatment differences by LSD test (*P* < 0.05). *, ** represents a significant difference at the levels of 0.05 and 0.01, respectively; ns represents no significant difference.

The fluctuations in leaf antioxidant enzyme activities (SOD, POD, and CAT) under different treatments are shown in Fig 6. The results of ANOVA indicated that CS, SFSF, and their interaction all had significant effects (*P* < 0.05) on CAT activity, while these factors had no significant effects on POD activity. CS and the interaction had significant affection SOD activity (*P* < 0.05).

Under “reclaimed-brackish water” cycling irrigation, the CAT and SOD activities in silicon fertilizer treatments rose with the increasing spraying frequency, which became statistically significant at 4 days of spraying frequency. Under RW-RW-BW cycling irrigation, both CAT and SOD activities initially rose and then decreased with the increasing spraying frequency, reaching the highest value at 2 days of silicon fertilizer spraying frequency. Similarly, POD activity followed a pattern of initial increase followed by a decrease, with the maximum value observed at two days of spraying frequency, which was significantly higher than the untreated control. Therefore, an appropriate increase in silicon fertilizer had an overall effect on leaf enzyme activity.

**Fig 7:**
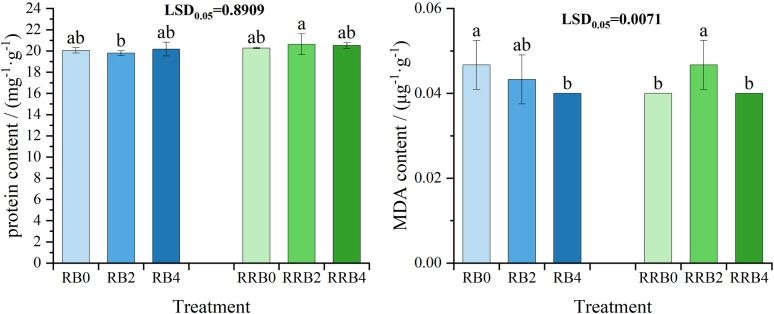
The interaction effect of CS*SFSF on alterations in leaf Malondialdehyde (MDA) and soluble protein levels subjected to cycling irrigation (CI) methods in conjunction with silicon nutrient application. Data are presented as means ± standard errors. Different lowercase letters on the column indicate significant treatment differences by LSD test (*P* < 0.05); *, ** represents a significant difference at the levels of 0.05 and 0.01, respectively; ns represents no significant difference.

Fig 7 showed the changes in MDA and soluble protein contents in different silicon fertilizer treatments under cycling irrigation. Under “reclaimed-brackish water” cycling irrigation, the MDA content of leaves treated with silicon fertilizer gradually decreased as the frequency of silicon fertilizer application increased, and the difference reached a significant level at 4 days of spraying frequency. In the case of RW-RW-BW cycling irrigation, the MDA content of leaves initially rose before declining as the application frequency prolonged, reaching the highest value in the spraying frequency of 2 days.

Under different cycling irrigation modes, there was no significant difference in the soluble protein content of leaves between those that received silicon fertilizer treatments and those that did not. In particular, leaves treated with silicon fertilizer every four days had a slightly higher soluble protein content than that of leaves not subjected to silicon fertilizer under “reclaimed-brackish water” cycling irrigation. Leaves that received silicon fertilizer exhibited a greater soluble protein content than those that did not under “reclaimed-reclaimed-brackish water” cycling irrigation. These findings indicate that the silicon fertilizer applied can lead to an increase in leaf-soluble protein content to a certain extent.

### Dispersion of silicon within plant-soil ecosystems under CI

**Fig 8:**
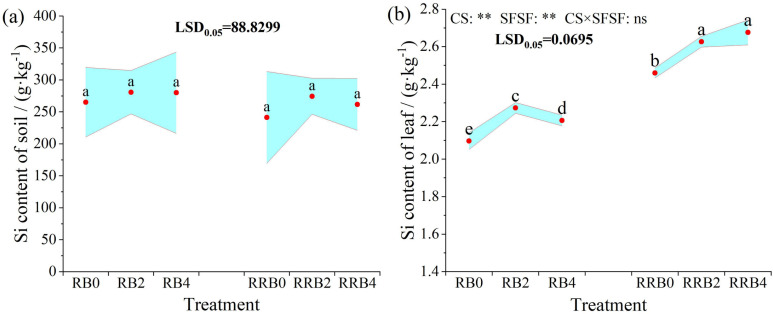
The interaction effect of CS*SFSF on fluctuations in silicon content within soil and leaf under cycling irrigation (CI) coupled with Si fertilization. Data are presented as means ± standard errors. Different lowercase letters on the column indicate significant treatment differences by LSD test (*P* < 0.05). *, ** represents a significant difference at the levels of 0.05 and 0.01, respectively; ns represents no significant difference.

Fig 8 illustrates the alterations in total silicon content in soil and leaves across. In comparison to the control group without silicon fertilization, the soil’s total silicon content was augmented by 5.64%-5.87% and 8.39%-13.68%, respectively under different cycling irrigation modes. However, these increases were statistically insignificant. Additionally, applying silicon fertilizer via leaf spraying did not considerably impact the soil’s total silicon content.

The results of ANOVA indicated that both AS and SFSF had significant effects on leaf silicon content (*P* < 0.05), but their interaction had no significant effect on leaf silicon content. Compared to the absence of silicon fertilizer, the total silicon content in leaf treated by Si-fertilizer significantly increased and reached the highest at 2 days of spraying frequency under “reclaimed-brackish water” cycling irrigation. Under “reclaimed-reclaimed-brackish water” cycling irrigation, the total silicon content in leaf treated progressively rose with an increase in spraying frequency. The silicon-fertilizer-treated leaves had a significantly higher silicon content than those untreated with silicon fertilizer. Meanwhile, through comparative analysis, it was found that the leaf silicon content under the “reclaimed-reclaimed-brackish water” cycling irrigation was significantly higher than that under the “reclaimed-brackish water” cycling irrigation. Suitable leaf surface application of silicon fertilizer enhances the total silicon content in plants, and irrigation further significantly increases leaf silicon content.

## Discussion

### How silicon supplementation influences soil properties under CI with BW and RW

The results of this study demonstrate that the EC in Si-fertilizer treatment was significantly greater compared to no Si-fertilizer treatment under “reclaimed-brackish water” cycling irrigation; however, under “reclaimed-reclaimed-brackish water” cycling irrigation, the soil EC at spraying frequency for 4 days was notably reduced compared to scenarios without silicon fertilizer application. This observation could be attributed to the progressive reduction in soil salinity as the proportion of reclaimed water under cycling irrigation increases. This finding aligns with existing research indicating that an increase in the use of reclaimed water leads to a decrease in EC [49]. In addition, the primary conclusion drawn from the data is that the pH values in all treatments ranged from 7.95 to 8.10, which remained comfortably below 8.5 and no threat of alkalinity; moreover, the soil ESP and SAR varied between 8.16% ~ 13.47% and 2.97 ~ 4.40 mM^1/2^, respectively, and were well below the threshold range (15% and 13 mM^1/2^), indicating no risk of soil salinization. Ci [50] found that adding exogenous mineral silicon had negligible influences on the rhizosphere soil moisture content. Similar results were obtained in our experiment; that is, the difference in soil moisture content was not significant with the extension of the silicon fertilizer spraying frequency under cycling irrigation. Likewise, in this experimental study, no substantial difference in soil moisture content was observed when the frequency of silicon fertilizer application was varied under a cycling irrigation regime.

Previous research has shown that silicon enhances the accumulation of potassium (K^+^) while simultaneously reducing sodium (Na^+^) levels within plants [51–53]. The results of this study demonstrate that, compared with no silicon fertilizer use, the soil K^+^ content was the highest under the silicon fertilizer spraying frequency for 4 days, and the soil Na^+^ content was the lowest under the silicon fertilizer spraying frequency for 2 days. Therefore, it was speculated that silicon could alleviate the ion imbalance caused by salt stress by adjusting the absorption, transport, and distribution of Na^+^. However, whether the sodium ions contained in silicon fertilizer have a regulatory effect on irrigation water still needs to be verified. After applying the silicon fertilizer, the soil Cl^-^ content first decreased and then increased under “reclaimed-brackish water” cycling irrigation, and the lowest was observed at the 2-day spraying frequency, aligning with prior findings [54,55]. Under “reclaimed-reclaimed-brackish water” cycling irrigation, the Cl^-^ content exhibited a trend of increasing-decreasing, and the lowest was found at 4 days of spraying frequency, which could be attributed to the rising percentage of reclaimed water usage and the steady decline in soil salinity [56], although the difference was not statistically significant. HCO_3_^-^ is the main ion that causes alkali stress in saline-alkali soil [57]. Our findings suggest that a steady decline in the soil HCO_3_^-^ concentration following the application of silicon fertilizer. According to the precipitation theory [58], an elevation in soil Ca^2+^ level triggers the production of additional CaCO_3_, consequently reducing the HCO_3_^-^ content, which is the opposite of the change in the total salt content.

### Effects of exogenous silicon on plant development and antioxidant indices under CI

Investigations have indicated that adding exogenous silicon is beneficial to plant growth under saline-alkali stress [59]. This research revealed that under “reclaimed-reclaimed-brackish water” cycling irrigation, the AFW slightly increased and reached the highest at 2 days of spraying frequency; under “reclaimed-brackish water” cycling irrigation, both the UFW and the UDW increased and reached the highest levels at 4 days of spraying frequency, which aligned with the relevant research outcomes of subsequent researchers [60–64]. Pati et al. [60] found that applying exogenous silicon could improve the crop yield of rice. Das et al. [61] found that applying exogenous silicon enhanced the organic acid level and enzyme activity in two varieties of rice plants, and improved their tolerance to salt stress. Kun [62] found that the incorporation of exogenous silicon in a culture solution could improve the drought resistance of tomato plants. Jam et al. [63] found that exogenous silicon application could improve the salt tolerance of safflower to some extent. Li et al. [64] showed that an appropriate amount of silicon fertilizer was conducive to improving the effective number of panicles per unit area, the number of grains per panicle, the seed setting rate, and ultimately, the total yield.

Abd-El-Aty et al. [65] found that the use of exogenous silicon fertilizer led to an increase in the levels of chlorophyll a, chlorophyll b, and carotenoids. Similarly, Rahmawati and Yasvi [66] observed a significant rise in chlorophyll a and b contents, as well as total chlorophyll, upon the application of silicon. Chen and Kattab [67] also reported an enhancement in seed yield and chlorophyll content resulting from the application of silicon fertilizer. Ahmed et al. [68] showed that silicon application enhanced the growth of rice plants and increased the levels of chlorophyll a and chlorophyll b, suggesting an improvement in photosynthetic efficiency. Our findings suggest that the changes in chlorophyll a, chlorophyll b, and total chlorophyll contents were the same, and they all decreased first and then increased as the frequency of silicon fertilizer spraying was prolonged; the highest was reached at 4-day spraying frequency. It can be concluded that silicon fertilizer may promote photosynthesis by increasing leaf area and chlorophyll content to promote photophosphorylation [69].

In this experimental study, it was observed that the CAT and SOD activities of the leaves treated with silicon fertilizer increased as the frequency of silicon fertilizer application progressed under “reclaimed-brackish water” cycling irrigation, while the CAT and SOD activities reached the highest when spraying frequency was 2 days under “reclaimed-reclaimed-brackish water” cycling irrigation. POD activity reached the highest at 2 days of spraying frequency under different cycling irrigation modes. Similarly, Dar et al. [70] discovered that treating buckwheat plants with exogenous silicon significantly regulated physiological and antioxidant reactions, overcame drug damage, and provided beneficial effects. Ahmed et al. [68] reported that silicon treatment led to the upregulation of the antioxidant enzymes, such as SOD, POD and CAT. Iqbal et al. [71] observed that silicon fertilizer significantly increased photosynthesis, leaf greenness, antioxidant enzyme activity, and carbohydrate metabolism in citrus plants. Chanthini et al. [72] found that applying exogenous silicon benefited salt-stressed plants by enhancing the activities of antioxidant enzymes in tomato plants. An appropriate use of silicon fertilizer had an overall impact on leaf enzyme activity. It can be concluded that the application of silicon fertilizer changed plant morphology and increased the activity of several key antioxidant enzymes (e.g., SOD) [73].

Yan et al. [74] demonstrated that silicon alleviated the excessive accumulation of malondialdehyde in rice plants caused by salt stress and boosted their soluble protein levels. Moukhtari et al. [75] reported that silicon eases oxidative stress in alfalfa exposed to salt stress by decreasing hydrogen peroxide and malondialdehyde concentrations and simultaneously enhancing proline accumulation. Mousavi et al. [76] showed that leaf spraying of silicon increased the levels of soluble protein and the activities of SOD, POD, and CAT, alleviating the detrimental impacts of salt-alkali stress on the physiological properties of cucumber plants. Similarly, the results of this study demonstrate that the MDA content of leaves gradually decreased with the extension of spraying frequency; Specifically, the soluble protein content in leaves subjected to a spraying interval of 4 days was marginally higher compared to the control without silicon fertilizer under “reclaimed-brackish water” cycling irrigation. Furthermore, the leaves treated with silicon fertilizer exhibited a higher soluble protein content than those without silicon treatment. under the “reclaimed-reclaimed-brackish water” cycling irrigation.

However, contrary to the findings of the previous paper [77] that had a similar experimental setup to this one, mixed solution of RW-BW in the previous paper vs alternating RW-BW irrigation combined with the same silicon fertilizer spray frequencies, but the results of the two papers showed different trends, such as EC, K, Cl, WDPT, pH, AFW, etc.

This is mainly because brackish water has a higher salt content than reclaimed water. Still, the overall salt concentration is diluted after mixing, and the total ion concentration is reduced. At the same time, the EC is positively correlated with the ion concentration in water, so the electrical conductivity value is reduced. In cycling irrigation, the sequence of reclaimed water-brackish water cannot wash away these salts in time. With the increase of irrigation times, the salts gradually accumulate in the soil [78], so the ionic concentration in the soil solution increases and the EC value rises. Mixed irrigation may trigger ion exchange or chemical reactions in which other ions on the colloidal surface of the soil interact with K^+^ and Cl^⁻^, absorbing part of K^+^, and Cl^⁻^ and leading to a reduction in the concentration of K^+^, and Cl^⁻^ in soil solutions. In cycling irrigation, the alternating dry and wet changes in soil moisture after each irrigation promote the weathering and dissolution of potassium-containing minerals in the soil, and release more K^+^ into the soil solution [79]. Mixed irrigation may change the wettability of the soil particle surface, reducing soil repulsion and allowing water droplets to penetrate the soil surface more easily. At the same time, the change of the chemical composition of the mixed water may change the dispersion or agglomeration of soil particles, forming a pore structure that is more conducive to water infiltration, thus shortening the WDPT. Cycling irrigation leads to large fluctuations in soil water content and salt content, which may make the dispersion and agglomeration of soil particles unstable, forming some pore structures that are not conducive to water infiltration. At the same time, the accumulation of salt may increase the repulsion of the soil, making it difficult for water droplets to penetrate the soil surface [78], increasing WDPT, etc.

## Conclusions

Based on a one-crop-cycle pot experiment, applying silicon (Si) fertilizer under cycling irrigation systems can positively influence soil properties and crop performance. Key findings indicate that Si fertilization effectively reduces soil sodium (Na⁺) and chloride (Cl⁻) concentrations, enhances the potassium-to-sodium (K/Na) ratio, and lowers the exchangeable sodium percentage (ESP) and sodium adsorption ratio (SAR), thereby reducing the risk of soil salinization. Optimal results were achieved with a spraying frequency of 2 days under “reclaimed-brackish water” cycling irrigation, which minimized ESP and SAR values, and led to a slight improvement in AWF.

However, this study is limited to a single crop cycle, and the results may not fully reflect the long-term effects of Si fertilization under reclaimed-brackish water irrigation. To validate these findings, long-term multi-crop-cycle experiments are essential to assess the sustained effects of Si fertilization under recycled irrigation systems. Future studies should also explore the optimal Si application rates and frequencies for different crops and soil types, as well as investigate the mechanisms underlying Si’s role in improving soil structure and crop resilience under saline conditions. Addressing these gaps will provide a more comprehensive understanding of Si’s potential in sustainable agricultural practices.
